# Intersexual and body size-related variation in chemical constituents from feces and cloacal products involved in intraspecific communication of a fossorial amphisbaenian

**DOI:** 10.7717/peerj.15002

**Published:** 2023-03-23

**Authors:** José Martín, Gonzalo Rodríguez-Ruiz, José Javier Cuervo, Pilar López

**Affiliations:** Department Ecología Evolutiva, Museo Nacional de Ciencias Naturales, CSIC, Madrid, Spain

**Keywords:** Amphisbaenians, Chemical Ecology, Communication, Lipids

## Abstract

**Background:**

Many animals rely on chemical cues for intraspecific communication. This is especially important in fossorial animals because visual restrictions of the underground environment limit the opportunities for visual communication. Previous experiments showed the ability of the amphisbaenian *Trogonophis wiegmanni* to discriminate between several categories of conspecifics based on chemical cues alone. However, in contrast with many other reptile species, *T. wiegmanni* does not have external secretory glands, but uses uncharacterized secretions from the cloaca in intraspecific chemosensory communication.

**Methods:**

Using gas chromatography-mass spectrometry (GC-MS), we analyzed the lipophilic compounds from feces and cloacal products freshly extracted from the cloaca of male and female *T. wiegmanni*. We identified and estimated relative proportions of the compounds found, and tested for intersexual and body-size related differences.

**Results:**

We found a total of 103 compounds, being some steroids (mainly cholesterol and cholestanol), some alkanes and squalene the most abundant and frequent. Further, we found intersexual differences, with males, especially larger ones, having higher proportions of several alkanes between C_13_ and C_24_ and of squalene than females, which had higher proportions of several steroids and also of nonacosane and methylnonacosane than males. We compared these findings with secretions of other animals and discuss the potential role of these compounds and their variations in intraspecific communication of amphisbaenians.

## Introduction

Chemical cues play a very important role in intra- and interspecific communication of many animal species (Wyatt, 2014). This is often based on specific compounds secreted from the skin or produced by specialized external glands. For example, in many reptiles, compounds from external femoral or precloacal gland secretions seem to be the basis of intraspecific communication (Mason, 1992; Weldon, Flachsbarth & Schulz, 2008; Mason & Parker, 2010; Martín & López, 2011, 2014). In addition, in many animals, different semiochemicals, secreted by internal glands are incorporated into feces or urine and used in communication (Müller-Schwarze, 2006). Reptiles are particularly interesting because in many diverse species of reptiles, fecal pellets that likely contain secretions from internal cloacal glands can provide detailed chemical information to conspecifics (*e.g*., Duvall, Graves & Carpenter, 1987; López, Aragón & Martín, 1998; Bull, Griffin & Perkins, 1999; Bull, Griffin & Johnston, 1999; Bull et al., 2001; Wilgers & Horne, 2009). These studies are a strong evidence that chemical signaling *via* fecal/cloacal secretions is a conserved trait in at least lizards, with an important role in their social and reproductive behavior (Mason, 1992; Martín & López, 2011). However, the chemical ecology (chemical composition and ecological roles) of these fecal and cloacal glandular secretions has been much less studied in reptiles (*e.g*., Bull, Griffin & Perkins, 1999) than, for example, in mammals (*e.g*., Müller-Schwarze, 2006; Martín, Barja & López, 2010; Miyazaki et al., 2018).

Amphisbaenians are a group of fossorial reptiles with characteristic morphological and functional adaptations (*e.g*., loss of limbs, narrow heads and reduced vision) that allow an underground lifestyle (Gans, 1974, 1978; Navas et al., 2004; Baeckens et al., 2017a). Given that fossoriality clearly limits the utility of visual cues, and that amphisbaenians have only very rudimentary vision (Gans, 1978), chemical senses seem prominent in these animals. Although only a few species have been examined, amphisbaenians use chemical cues to identify their prey and predators (López & Martín, 1994, 2001; Semhan, Halloy & Montero, 2010; López, Ortega & Martín, 2014), and to discriminate between classes of conspecifics (Cooper, López & Salvador, 1994; López & Martín, 2009; Martín et al., 2020; Martín et al., 2021).

The checkerboard worm lizard *Trogonophis wiegmanni* is a strictly fossorial amphisbaenian found in sandy soils in NW Africa (Bons & Geniez, 1996; Martín, López & García, 2013). Previous studies measuring tongue-flicking behavior showed that this amphisbaenian is able to discriminate conspecifics using chemical cues from the cloaca (Martín et al., 2020). At least male amphisbaenians recognized the sex of unfamiliar conspecifics based on scent alone. Moreover, both males and females discriminated between a familiar partner and an unfamiliar individual of the same sex as the partner, and males discriminated between self and unfamiliar male scents (Martín et al., 2020). Further, adult females, but not males, could discriminate between the scents of familiar juveniles, likely their offspring, and unfamiliar ones (Martín et al., 2021). Therefore, these behavioral studies suggest that cloacal chemical cues provide information on the sexual and individual identity of this amphisbaenian.

However, the chemical basis of such chemosensory abilities of amphisbaenians are practically unknown. In other amphisbaenian species, *Blanus cinereus*, sex discrimination seems to be based on variation of some lipophilic compounds secreted by the external precloacal glands, such as squalene, fatty acids, cholesteryl methyl ether and cholesterol (López & Martín, 2005; López & Martín, 2009). Precloacal glands, however, are not found in *T. wiegmanni*, although it has internal cloacal glands (Bons & Saint Girons, 1963). We hypothesized that similar lipophilic compounds in secretions coming from these cloacal glands might be incorporated into fecal products and be the basis of chemosensory communication in this amphisbaenian.

In this article, we analyzed using gas chromatography-mass spectrometry (GC-MS) the lipophilic compounds from fecal and cloacal products freshly and directly extracted in the field from the cloaca of male and female amphisbaenians *T. wiegmanni*. We identified and estimated relative proportions of the compounds found, tested for intersexual and body size-related differences, and discuss the potentiral role of these compounds in intraspecific communication of this amphisbaenian.

## Materials and Methods

### Field sampling of feces and cloacal products of amphisbaenians

During June 2022, we conducted field work at the Chafarinas Islands (35°11′N, 2°25′W; Spain), where *T. wiegmanni* is very abundant (Martín et al., 2011a, 2011b). This is a small volcanic archipelago located in the southwestern area of the Mediterranean Sea, 4.6 km offshore the northern Moroccan coast (Ras el Ma, Morocco). The archipelago consists of three small islands: Congreso (25.6 ha), Isabel II (15.1 ha; the only one inhabited by humans) and Rey Francisco (13.9 ha).

We looked for amphisbaenians under stones in Isabel and Rey Island and captured individuals by hand. Fecal and cloacal samples were obtained from live amphisbaenians immediately after capture in the field. Amphisbaenians usually defecated gastrointestinal contents when handled, but we sometimes needed to compress gently their vents to force the expulsion of feces. We used 1.1 ml total recovery chromatography glass vials (ref. V2275; Análisis Vínicos S.L., Tomelloso, Spain) to individually store a small amount of each fecal sample and associated liquid products directly taken from the cloaca. Vials were closed with a Teflon-lined stopper and a screw cap, labeled and temporally kept in a refrigerated box in the field and later stored in a freezer at −20 °C until analyses were performed 1 month after. We also made blank control vials using the same procedure but without collecting feces, which were treated and analyzed in the same manner to compare with the fecal samples and to be able to detect potential contaminants from the handling or analytical procedure.

We determined the sex of amphisbaenians by carefully everting the hemipenes of males from the cloacas (Martín et al., 2011b) and measured their snout-to-vent length (SVL) using a metal ruler. Males and females sampled were adults that did not significantly differ in SVL (males, mean ± SE = 136 ± 9 mm, range = 83–174 mm, *n* = 11; females, 146 ± 6 mm, range = 124–176 mm, *n* = 9; One-way ANOVA on log-transformed SVL, *F*_1,18_ = 0.99, *p* = 0.33). Animals were released to their capture sites in a few minutes. We ensured that the same individuals were not sampled twice because amphisbaenians were marked individually with PIT-tags as part of a long-term population study (Recio et al., 2019).

The captures enforced all the present Spanish laws and were performed under license granted by the “Organismo Autónomo de Parques Nacionales” (Spain) (n° 12706) and in accordance with the national animal welfare standards and protocols supervised by the “Comisión Ética de Experimentación Animal (CEEA)” (Ethical Committee of the National Museum of Natural Sciences, Spanish Research Council, CSIC) (Ref. 901/2020).

### Chemical analyses of compounds in feces

In the laboratory, we thawed the vials with samples and added 250 μl of n-hexane (capillary GC grade; Sigma-Aldrich Chemical Co, Saint Louis, MO, USA) to each vial. The vial was closed and we mixed the solution for 1 min using a vortex. Thereafter, the vial was placed in a fridge for 10 min to rest until the solid material that was not dissolved precipitated at the bottom of the vial. We extracted the supernatant clear liquid phase with a glass syringe and transferred it to a clean vial that was closed with a Teflon-lined stopper.

To analyze samples, we used a gas chromatograph (GC) (Agilent 7890A; Agilent, Santa Clara, CA, USA) coupled to a mass spectrometer (MS) (Agilent 5975C with Triple-Axis HED-EM detector). The GC was equipped with a poly (5% phenyl/95% methylpolysiloxane) Agilent HP5-MS column (30 m length, 0.25 mm ID, 0.25 mm film thickness). The oven of the GC was programmed so that the temperature was held initially at 45 °C for 10 min, and then increased at a rate of 5 °C/min until a final temperature of 280 °C, which was held for 20 min. We used helium at 0.6 ml/min as the carrier gas. We injected 2 µl of each sample in splitless mode with an inlet temperature of 255 °C. Ionization by electron impact (70 eV) was carried out at 150 °C with a source temperature of 230 °C. We did not record mass spectral fragments below *m/z* = 28.

The initial tentative identification of the compounds found in the fecal samples was carried out by comparing the fragmentation patterns (*i.e*., mass spectra) of the compounds detected in the samples with those available in the NIST/EPA/NIH 2002 computerized mass spectral library. When possible, the identification was confirmed by comparing the spectra and retention times with those obtained under the same analytical conditions of the analysis using authentic standards (from Sigma-Aldrich Chemical Co) (see Table S1). Impurities identified in the control vial samples (*e.g*., hydroperoxyhexanes, siloxanes, phthalates, *etc*.) are not reported.

### Data analyses

The relative amount of each chemical compound was determined as the compound peak area in the chromatogram divided by the total peak area (TIC area), excluding contaminants, and multiplied by 100. For this, we used the integration capacity of the peak areas available in the software Xcalibur (Finningan Co., Saranac Lake, NY, USA). For statistical analyses, the relative proportions of each compound were transformed following the formula: ln ((proportion)/(1- proportion)), to correct the problem of non-independence between proportions (Aebischer, Robertson & Kenward, 1993; García-Roa et al., 2018).

To test for intersexual differences in the chemical profiles, we used the software PRIMER V6.1.13 (Clarke & Gorley, 2006) and PERMANOVA + V1.0.3 (Anderson, Gorley & Clarke, 2008). Analyses were made considering all the compounds found or restricted to the 20 most abundant compounds (*i.e.*, with the highest mean relative proportions considering the average abundance across all individuals), and which frequencies of appearance were also high (mean = 63%; range = 35–100%). We first calculated the Euclidean distances between every pair of individual samples and produced a resemblance matrix that was the basis for further analyses. Then, to compare the chemical profiles between sexes, we used permutational multivariate variance analyses (PERMANOVA, based on the Euclidean resemblance matrix and using 999 permutations) (Anderson, 2001), and canonical analyses of principal coordinates (CAP) (Anderson & Willis, 2003).

The transformed areas of the 20 most abundant compounds were also used to make a principal component analysis (PCA). The factor scores of the extracted principal components (PCs) were used as new dependent variables in general linear models (GLM) to test for differences between sexes (fixed factor) and in relationship with SVL (log_10_-transformed, continuous factor) and including the interactions sex x SVL in the models. When an interaction was significant, we calculated separately for males and females Spearman’s rank order correlations between PC scores and log_10_-transformed SVL to explore the meaning and direction of such interaction. Statistical analyses were performed using the software Statistica 7.0 (StatSoft Inc., Tulsa, OK, USA).

## Results

### Chemicals in feces and cloacal secretions of adult amphisbaenians

We found a total of 103 lipophilic compounds in fresh feces and cloacal secretions collected directly from adult amphisbaenians (Table S1). However, the number of compounds detected in a single individual was much lower, ranging between 10 and 54 (mean ± SD = 33 ± 13 compounds/fecal sample). Most of the major compounds were found in most of the samples (*i.e*., 18 compounds appeared in at least half of the samples with a mean proportion of 3.8%), whereas other minor compounds were found only occasionally (*i.e*., 85 compounds appeared in less than half of the samples with a mean proportion of 0.4%) (Table S1).

The main compounds were 34 steroids (population mean ± SD = 53.90 ± 24.07% of the TIC area), 22 linear alkanes between *n*-C_12_ and *n*-C_36_ (18.86 ± 14.18%), 21 branched alkanes (13.13 ± 11.87%) and one terpenoid (squalene, 9.57 ± 13.81%). In addition, we also found other minor compounds, such as eight alcohols (3.14 ± 5.07%), three aromatic heterocyclic compounds (0.72 ± 0.95%), nine methyl esters of carboxylic acids between *n*-C_14_ and *n*-C_18_ (0.35 ± 0.69%), one ketone (0.17 ± 0.48%), three aldehydes (0.14 ± 0.36%), and cyclic octaatomic sulfur (<0.01%) (Table S1). On average, pooling all adult individuals, the five most abundant compounds were cholesterol (11.4%), cholestan-3β-ol (10.3%), squalene (9.6%), cholest-5-en-3-ol acetate (8.1%) and 11-methylnonacosane (6.7%), although these proportions varied between sexes (see below).

### Intersexual and body size-related differences

**Figure 1:**
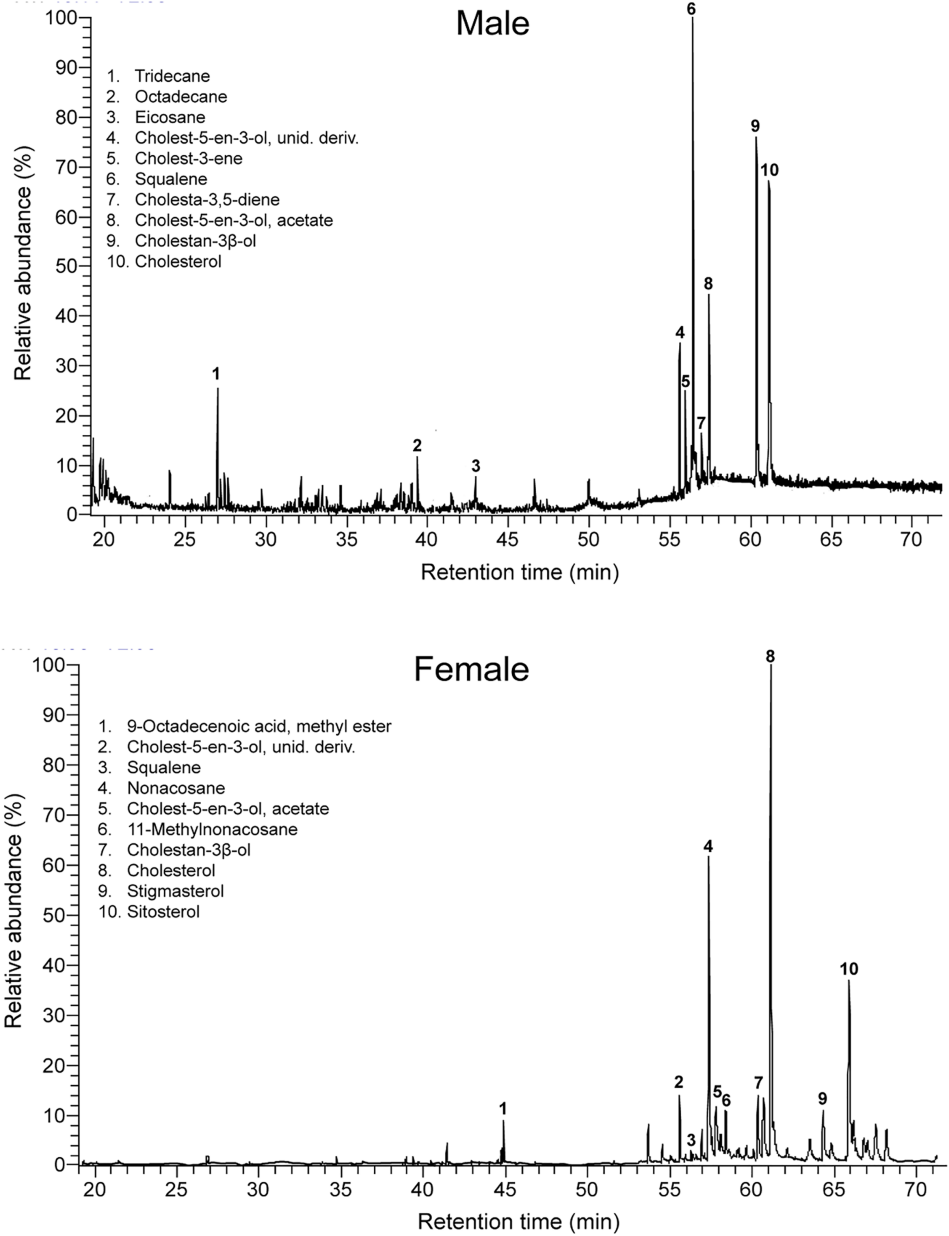
Intersexual differences in compounds in feces of amphisbaenians. Representative gas chromatogram traces of a male (top) and a female (bottom) individual amphisbaenian *T. wiegmanni*. Numbers above some peaks refer to characteristic compounds listed inside each graph.

**Figure 2:**
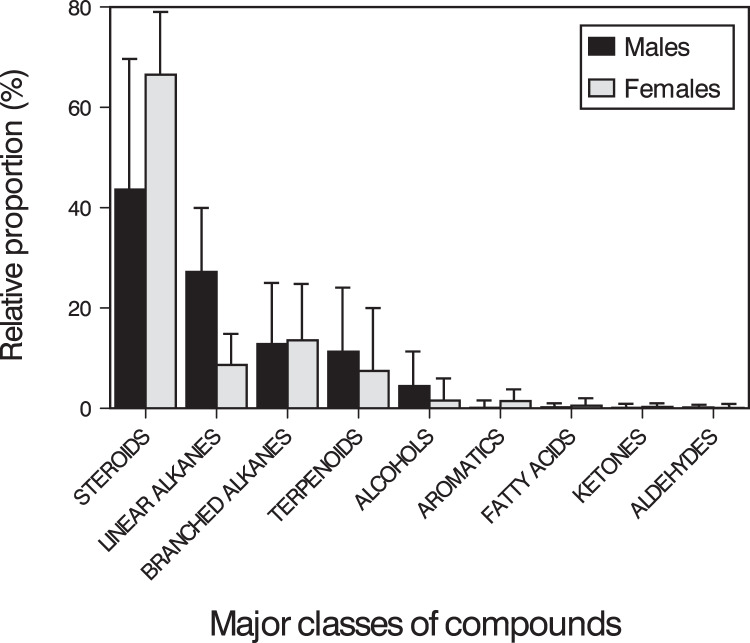
Classes of compounds in feces of amphisbaenians. Mean (+ SD) relative proportions (% of TIC area) of the major classes of compounds found in feces and cloacal products of males (black bars) and females (grey bars) of the amphisbaenian *T. wiegmanni*.

Males and females had similar types of lipophilic major compounds and a similar total number of compounds in a single sample (males: mean ± SD = 32 ± 9; females: 34 ± 17; GLM on log_10_-transformed number of compounds, sex, *F*_1,16_ = 0.45, *p* = 0.51), and this number was not significantly related with body size (SVL, *F*_1,16_ = 0.72, *p* = 0.41; sex x SVL, *F*_1,16_ = 0.39, *p* = 0.54). However, there were some intersexual differences in the GC traces (Fig. 1). In particular, there were significant differences between males and females in the proportions of the major classes of compounds (Pearson’s *χ*^*2*^ = 12.83, *p* = 0.025) (Fig. 2) and in the rank order of the main compounds; the five most abundant compounds of males were squalene (11.2%), cholestan-3β-ol (10.9%), cholesterol (9.3%), cholest-5-en-3-ol acetate (6.7%) and cholest-3-one (3.9%), whereas the main compounds of females were cholesterol (13.8%), 11-methylnonacosane (10.5%), cholest-5-en-3-ol acetate (9.9%), cholestan-3β-ol (9.6%) and squalene (7.5%) (Table S1). The PERMANOVA analysis based on the resemblance matrix comparing samples of each sex showed significant differences in the overall proportion of compounds between males and females (pseudo *F*_1,18_ = 5.35, *p* = 0.004). The CAP analysis assigned 80% of the chemical profiles into the correct sex using the Euclidean distances between samples (permutational test, *δ*_20_ = 0.97, *p* = 0.02, using leave-one-out cross-validation and *m* = 11 axis). Similar analyses restricted to the 20 most abundant compounds yielded similar results (PERMANOVA, pseudo *F*_1,18_ = 6.25, *p* = 0.004; CAP, 80% correct assignations; permutational test, *δ*_20_ = 0.81, *p* = 0.019, *m* = 10 axis). Interestingly, the three individual males, which sex was incorrectly assigned by the CAP based on their chemical profiles, were those with the smallest SVL.

**Figure 3:**
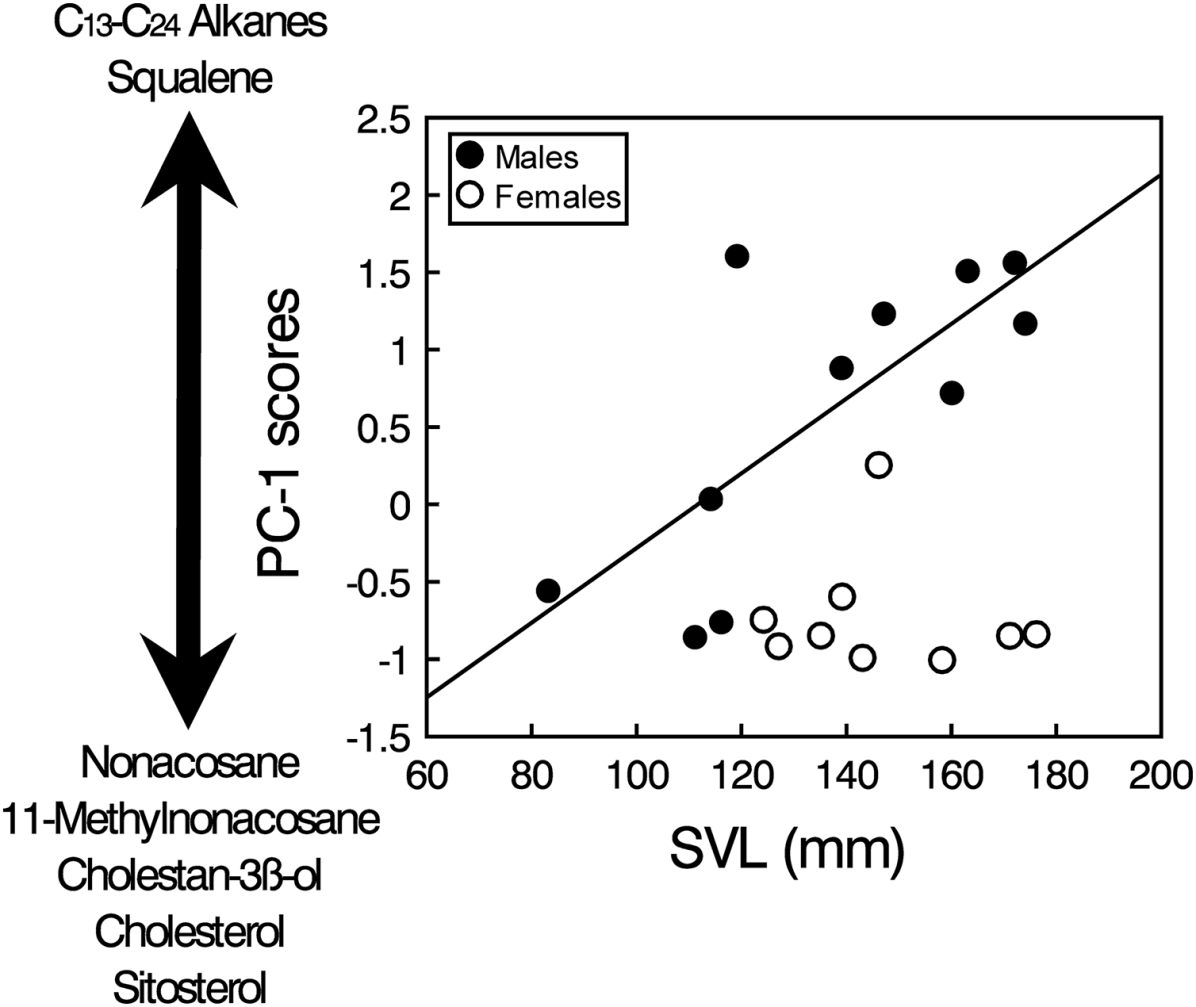
Sex and body size-related variation in compounds in feces of amphisbaenians. Relationship between body size (SVL) of males (black circles, continuous line) and females (white circles) of the amphisbaenian *T. wiegmanni* and the PC-1 scores from a PCA describing the profile of compounds found in their feces and cloacal products. The arrow indicates the correlations of the PC-scores with the relative proportions of determined compounds.

The PCA analysis of the transformed areas of the 20 most abundant compounds extracted four principal components (PCs) with eigenvalues greater than one, which together accounted for 86.2% of the variance (Table 1). The analysis of the PC scores resulting from the PCA showed that there were significant intersexual differences in the compounds described by PC1 (GLM, sex: *F*_1,17_ = 10.33, *p* = 0.0051), which were not significantly related to body size when pooling all individuals (SVL: *F*_1,17_ = 0.08, *p* = 0.78), but the interaction was significant (sex x SVL: *F*_1,17_ = 11.51, *p* = 0.0035). Analyzing the two sexes separately, the relationship between PC1 and SVL was positive and significant in males (Spearman’s correlation, *r*_*s*_ = 0.67, *t* = 2.73, *p* = 0.023), but there was not any significant relationship in females (*r*_*s*_ = 0.02, *t* = 0.04, *p* = 0.97) (Fig. 3). Thus, according to correlations of the compounds with the PC1 (Table 1), males, especially larger ones, had higher proportions of several alkanes between C_13_ and C_24_ and higher proportions of squalene than females, which in contrast had higher proportions of several steroids such as cholesterol, cholestanol or sitosterol and also high proportions of nonacosane and methylnonacosane than males (Table S1). There were not significant differences between sexes or significant relationships with SVL in PC2 (GLM, sex: *F*_1,17_ = 0.21, *p* = 0.65; SVL: *F*_1,17_ = 0.01, *p* = 0.90; sex x SVL: *F*_1,17_ = 0.20, *p* = 0.66), PC3 (GLM, sex: *F*_1,17_ = 0.70, *p* = 0.41; SVL: *F*_1,17_ = 0.02, *p* = 0.880; sex x SVL: *F*_1,17_ = 0.69, *p* = 0.42) or PC4 (GLM, sex: *F*_1,17_ = 0.58, *p* = 0.46; SVL: *F*_1,17_ = 0.05, *p* = 0.83; sex x SVL: *F*_1,17_ = 0.53, *p* = 0.47).

**Table 1:** Principal components analysis (PCA) for the 20 compounds more abundant in feces of the amphisbaenian *Trogonophis wiegmanni*. Compound are presented in order of abundance. Correlations between variables (compounds) and the principal components that were significant at *p* < 0.01 are marked in bold. RT = retention time (min).

Compound	PC1	PC2	PC3	PC4
Cholesterol	**−0.69**	−0.48	0.21	0.37
Cholestan-3β-ol	**−0.59**	0.55	0.29	0.48
Squalene	**0.75**	−0.03	0.26	−0.20
Cholest-5-en-3-ol, acetate	−0.39	−0.38	**0.68**	−0.26
11-Methylnonacosane	**−0.81**	0.25	−0.07	−0.16
Cholest-5-en-3-ol, unidentified derivative	−0.28	**−0.64**	**0.67**	0.02
Nonacosane	**−0.80**	0.16	−0.42	0.11
Cholestan-3-one	0.04	**0.91**	0.13	−0.13
Cholest-3-ene	−0.15	**0.76**	0.37	0.18
Sitosterol	**−0.76**	−0.32	−0.34	0.27
Unidentified branched alkane at RT 58.1	**−0.63**	0.34	0.04	−0.47
Octadecane	**0.95**	−0.05	−0.07	0.18
Eicosane	**0.90**	−0.10	0.06	0.22
Docosane	**0.95**	0.01	−0.04	0.15
Cholesta-3,5-diene	−0.13	**−0.84**	0.25	0.00
Tridecane	**0.92**	−0.06	0.01	0.19
Unidentified steroid at RT 58.4	**−0.92**	−0.23	−0.11	−0.01
Cholest-2-ene	−0.48	**0.65**	0.42	0.38
Tetracosane	**0.93**	−0.11	−0.01	0.17
Stigmastan-3,5-diene	**−0.76**	−0.46	−0.26	0.24
				
Eigenvalue	9.90	4.21	1.89	1.23
Explained variance (%)	49.52	21.03	9.47	6.14

## Discussion

Our chemical analyses showed the occurrence of a high diversity of lipophilic compounds, mainly steroids and alkanes, in feces of the amphisbaenian *T. wiegmanni*. Some of these compounds might have a role as semiochemicals in intraspecific communication, as suggested by previous behavioral experiments based on tongue-flicking assays that measured the rate of biochemical sampling to cloacal scents (Martín et al., 2020, 2021).

The consistent patterns of presence and abundance of some specific major compounds in feces of *T. wiegmanni* suggest that these compounds are not merely remains of the prey (*e.g*., insects and other invertebrates), which can vary between individual fecal samples (*e.g.*, feces containing mainly snails *vs*. those with mainly adult beetles). Thus, part of the compounds showing a consistent pattern across individuals might be secreted by cloacal glands and be incorporated into feces. Moreover, given that there are no intersexual differences in the diet of this species (Martín et al., 2013), the observed significant intersexual overall differences in proportion of compounds in feces and cloacal secretions also support that at least some of these compounds are not dependent directly on the prey remains contained in feces. Also in lizards, exocrine secretions coming from femoral or preclocacal glands are not directly produced by diet/prey components and, moreover, diet seems a relatively poor predictor of interspecific differences in the chemical profiles of femoral secretions of lizards (Baeckens et al., 2017b).

Similarly, in some skinks and lizard species, compounds with a semiochemical function are secreted onto the surface of the feces, probably coming from cloacal glands, as feces are deposited by the animal. These feces have been found to allow conspecific and sex discrimination in several skinks (Bull, Griffin & Perkins, 1999; Bull, Griffin & Johnston, 1999; Bull et al., 2000, 2001), lacertids (Aragón, López & Martín, 2000; Nisa-Ramiro et al., 2019), iguanians (Duvall, 1979, 1981; Duvall, Graves & Carpenter, 1987; Labra et al., 2002; Wilgers & Horne, 2009) and geckos (Carpenter & Duvall, 1995). Fecal pellets may even be used for territorial scent marking (Duvall, Graves & Carpenter, 1987; Carpenter & Duvall, 1995; López, Aragón & Martín, 1998). For example, in some Australian skinks (*Egernia striolata* and *E. stokesii*), individuals respond more strongly to chemicals from feces of unfamiliar conspecifics than to their own scats (Bull, Griffin & Perkins, 1999; Bull, Griffin & Johnston, 1999). This difference in behavior was unrelated to diet because there was no difference in the response to scats from unfamiliar lizards fed on the same or a different diet from the test skink. Specific compounds with semiochemical properties have not been identified in the feces of these skinks. However, these semiochemicals are probably a combination of several lipids that were contained in scat extracts made with organic solvents (dichloromethane), because further fractionation of the scats with different solvents (pentane and methanol) led to loss of the lipids and the unique signals needed for individual recognition (Bull, Griffin & Perkins, 1999).

Steroids are the major compounds found in feces of *T. wiegmanni* amphisbaenians, which coincides with the overall composition of femoral gland secretions of many lizards (Weldon, Flachsbarth & Schulz, 2008; Martín & López, 2014) and of precloacal gland secretions of the amphisbaenian *B. cinereus* (López & Martín, 2005, 2009). In most lizard species examined so far, cholesterol is often the main compound found in glandular secretions (Weldon, Flachsbarth & Schulz, 2008; Martín & López, 2014). Also, cholesterol is a major compound in feces of *T. wiegmanni*, although cholestanol is also similarly abundant. The latter is a metabolite of its biological precursor, cholesterol, produced by oxidation by symbiotic anaerobic microorganisms found in the digestive tract (Scheline, 1973). Therefore, similarly to lizards, it is likely that cholesterol might be the main compound in cloacal secretions of *T. wiegmanni*, although a large part of cholesterol would be later transformed to cholestanol in the cloaca by bacteria before being expelled in feces. Also, in birds, symbiotic bacteria in the uropygial gland are known to transform and produce volatile compounds that are behaviorally relevant for communication (Whittaker et al., 2019). In general, in vertebrates, the source specificity of fecal steroids is a combination of steroid intake, metabolic production of steroids and also the characteristics of the microbiota resident within the animal’s digestive tract (Leeming et al., 1996). Thus, as it occurs in lizards (Martín & López, 2015), variations in steroids in feces and cloacal secretions of *T. wiegmanni* amphisbaenians may indirectly reflect not only the sex, but also the metabolism, body size, condition and quality of the producer and be important in communication.

Alkanes were the second more abundant class of compounds found in feces of *T. wiegmanni*, which could be simply remains of prey. However, the higher abundance and diversity of alkanes found in males in comparison with females, in spite of the lack of dietary differences (Martín et al., 2013), suggests again that at least some of these alkanes might have a different source, perhaps from internal cloacal glands whose secretion might differ between sexes. In many insects, differences in cuticular or glandular hydrocarbon profiles allow interspecific and intersexual discrimination (Wyatt, 2014). Similarly, differences in alkane profiles might also have an important role in communication in *T. wiegmanni*, for example, explaining its reported self and familiar chemosensory recognition abilities (Martín et al., 2020, 2021). In this sense, the relative high abundance in feces of *T. wiegmanni*, especially in females, of two long-chain alkanes (heptacosane and nonacosane) and their derivative monomethylalkanes (11-methylheptacosane and 11-methylnonacosane) is particularly interesting for their potential in sex and familiar recognition. Similarly, many insects, such as some beetles and ants, utilize 11-methylnonacosane and similar branched alkanes in their chemical communication systems (*e.g*., Dahbi et al., 1996; Sugeno, Hori & Matsuda, 2006). For example, the mating behavior of male beetles *Gastrophysa atrocyanea* is elicited by the presence of these and similar cuticular monomethylalkanes on the surface of the female body (Sugeno, Hori & Matsuda, 2006). Also, multiple species of garter snakes and the brown treesnake produce in the skin blends of long-chain hydrocarbons (methyl ketones) that have a chemical signaling function (Mason et al., 1989; Mason et al., 1990; Parker et al., 2018) and similar long-chain lipids are also used by cockroaches for chemical communication (Eliyahu et al., 2008). Therefore, the blends of long-chain alkanes found in feces of *T. wiegmanni* might have a similar role in sex identification, and even in familiar recognition and reproductive behavior, which warrants further investigation.

Squalene seems also an important compound in the feces of *T. wiegmanni*, being the main single compound in males, which have higher proportions than females. Interestingly, in the precloacal gland secretions of the amphisbaenian *B. cinereus*, squalene also shows similar importance and intersexual differences, and this compound alone seems to allow chemosensory sex discrimination by males (López & Martín, 2005, 2009). Squalene has also been identified as one component of the male recognition system of garter snakes (*Thamnophis sirtalis*) (Mason et al., 1989). Thus, when squalene was experimentally supplemented on the skin of a female snake, the intensity of courtship to which this female was subjected was reduced (Shine et al., 2005). Furthermore, in amphisbaenians, squalene might also signal dominance status or aggressiveness of males because higher concentrations of squalene presented alone elicit higher levels of aggression by males in the amphisbaenian *B. cinereus* (López & Martín, 2009). The relationhsip between squalene and a signal of dominance might be explained because squalene is the biochemical precursor of many steroids, including steroid hormones such as testosterone, and there is likely some metabolic relationship between the circulating amounts of these two compounds. Similar roles might be predicted for the squalene found in feces and cloacal secretions of the amphisbaenian *T. wiegmanni*. In fact, we found that proportions of squalene, and of several alkanes too, were greater in larger males (see Fig. 3). Further experiments should examine responses of this amphisbaenian to squalene and alkanes with different concentrations, and look for empirical and experimental relationships between proportions of these compounds, body size, reproductive hormones and behavioral variables such as aggressiveness.

Finally, other minor compounds that are common in secretions of other reptiles were also found in feces of *T. wiegmanni*. However, these minor compounds appeared in very low proportions and in most cases only occasionally, which would not point to a potential role in communication in this amphisbaenian. For example, in contrast to exocrine secretions of lizards and the amphisbaenian *B. cinereus*, where fatty acids, such as hexadecanoic, octadecenoic and octadecanoic acid, are often very abundant and frequent (Weldon, Flachsbarth & Schulz, 2008; Martín & López, 2014), we only found occasional and very small amounts of several methyl esters of fatty acids in some feces of *T. wiegmanni*.

## Conclusions

We conclude that the presence and patterns of intersexual and body size-related differences in some compounds in feces of *T. wiegmanni* strongly suggest that these compounds, and their variations, might be the basis for explaining the already known intraspecific chemosensory responses to feces and cloacal secretions of this amphisbaenian species. Future studies should examine experimentally the behavioral responses to these specific compounds, or mixes of compounds, and explore the potential physiological relationships between chemical profiles and individual characteristics, which would help to maintain the reliability of these potential semiochemicals in intraspecific communication.

## Supplemental Information

10.7717/peerj.15002/supp-1 Supplemental Information 1 Lipophilic compounds found in hexane extracts of feces of male and female amphisbaenians *Trogonophis wiegmanni*. Relative proportion (mean + SD percent of the total ion current, TIC, of each compound in each fecal sample) and frequency (% of fecal samples containing a particular compound) are shown. An asterisk after the compound name indicates that the identification was confirmed with standards. The other compounds were tentatively identified based on mass spectra and retention times (RT). Characteristic ions (*m/z*) are reported for unidentified steroids (Un. Ster.).

## Additional Information and Declarations

### Field Study Permissions

The following information was supplied relating to field study approvals (*i.e*., approving body and any reference numbers): The captures enforced all the present Spanish laws and were performed under license granted by the “Organismo Autónomo de Parques Nacionales” (Spain) (n° 12706).

### Data Availability

The following information was supplied regarding data availability: The data is available at figshare: martin, jose; Rodriguez-Ruiz, Gonzalo; Cuervo, Jose Javier; López, Pilar (2022): Intersexual and body size variation in chemical constituents from feces and cloacal products involved in intraspecific communication of a fossorial amphisbaenian. figshare. Dataset. https://doi.org/10.6084/m9.figshare.21774497.v1.
