# Supplementary material for: Intersexual and body size-related variation in chemical constituents from feces and cloacal products involved in intraspecific communication of a fossorial amphisbaenian
Source: PeerJ. 2023 Mar 23;11:e15002. doi: 10.7717/peerj.15002 (PMC10040184; doi:10.7717/peerj.15002)
Supplement: Supplemental Information 1 — Relative proportion (mean + SD percent of the total ion current, TIC, of each compound in each fecal sample) and frequency (% of fecal samples containing a particular compound) are shown. An asterisk after the compound name indicates that the identification was confirmed with standards. The other compounds were tentatively identified based on mass spectra and retention times (RT). Characteristic ions (m/z) are reported for unidentified steroids (Un. Ster.). [file peerj-11-15002-s001.docx]

**Table S1.** Lipophilic compounds found in hexane extracts of feces of male and female amphisbaenians *Trogonophis wiegmanni.* Relative proportion (mean + SD percent of the total ion current,TIC, of each compound in each fecal sample) and frequency (% of fecal samples containing a particular compound) are shown. An asterisk after the compound name indicates that the identification was confirmed with standards. The other compounds were tentatively identified based on mass spectra and retention times (RT). Characteristic ions (*m/z*) are reported for unidentified steroids (Un. Ster.).

| RT  (min) | Compound | Males  (*n* = 11) | | | | Females  (*n* = 9) | | | |
| --- | --- | --- | --- | --- | --- | --- | --- | --- | --- |
|  |  | Proportion (%)  (mean ± SD) | | | Freq.  (%) | Proportion (%)  (mean ± SD) | | | Freq. (%) |
| STEROIDS: | |  |  |  |  |  |  |  |  |
| 55.59 | Cholest-5-en-3-ol, unidentified derivative | 3.72 | ± | 2.80 | 100 | 4.60 | ± | 3.42 | 100 |
| 55.93 | Cholest-3-ene * | 3.89 | ± | 5.08 | 73 | 3.57 | ± | 3.05 | 100 |
| 56.30 | Cholest-2-ene * | 1.25 | ± | 1.56 | 73 | 1.14 | ± | 0.84 | 100 |
| 56.56 | Cholest-4-ene * | 0.79 | ± | 0.79 | 64 | 0.30 | ± | 0.33 | 67 |
| 56.57 | Cholesta-2,4-diene * | 0.17 | ± | 0.42 | 18 | 0.27 | ± | 0.43 | 44 |
| 56.94 | Cholesta-3,5-diene * | 1.27 | ± | 1.22 | 73 | 2.14 | ± | 1.86 | 78 |
| 57.12 | Cholesta-4,6-dien-3-ol * | 0.20 | ± | 0.30 | 36 | 0.26 | ± | 0.34 | 67 |
| 57.42 | Cholest-5-en-3-ol, acetate * | 6.69 | ± | 3.47 | 100 | 11.07 | ± | 8.41 | 100 |
| 57.58 | Stigmasta-5,22-dien-3-ol, acetate | 0.12 | ± | 0.26 | 18 | 0.11 | ± | 0.23 | 33 |
| 58.40 | Un.Ster. (197,213,255,275 351,379,393,422) | 0.60 | ± | 1.03 | 27 | 2.07 | ± | 1.49 | 89 |
| 58.82 | Un.Ster. (201,215,257,275,325,383,398,422) | 0.02 | ± | 0.08 | 9 | 0.90 | ± | 1.39 | 67 |
| 59.15 | Un.Ster. (147,177,213,255,367,382,406,430) | 0.20 | ± | 0.59 | 18 | 0.19 | ± | 0.38 | 33 |
| 59.64 | Un.Ster. (147,215,255,282,343,398) | 0.08 | ± | 0.20 | 18 | 0.22 | ± | 0.36 | 56 |
| 60.04 | Un.Ster. (213,255,342,381,396,415,430) | 0.05 | ± | 0.12 | 18 | 0.09 | ± | 0.18 | 33 |
| 60.34 | Cholestan-3β-ol * | 10.91 | ± | 10.87 | 73 | 8.84 | ± | 4.53 | 100 |
| 60.70 | Stigmastan-3,5-diene | 0.74 | ± | 1.68 | 27 | 1.42 | ± | 1.56 | 67 |
| 61.13 | Cholesterol * | 9.33 | ± | 11.55 | 45 | 13.25 | ± | 11.63 | 78 |
| 61.16 | Cholestan-3-one * | 3.85 | ± | 8.50 | 27 | 3.62 | ± | 4.53 | 44 |
| 61.26 | Cholestan-3α-ol | 0.61 | ± | 1.11 | 27 | 1.01 | ± | 0.86 | 78 |
| 61.86 | Desmosterol * | 0.04 | ± | 0.13 | 9 |  | - |  |  |
| 62.12 | Ergosta-5,22-dien-3-ol |  | - |  |  | 0.12 | ± | 0.23 | 33 |
| 62.19 | Cholestan-3-one, unidentified derivative (429) | 0.05 | ± | 0.11 | 18 |  | - |  |  |
| 62.56 | Ergostanol * | 0.05 | ± | 0.12 | 18 | 0.09 | ± | 0.18 | 33 |
| 63.51 | Campesterol * | 0.23 | ± | 0.56 | 18 | 0.49 | ± | 0.74 | 56 |
| 64.06 | Cholest-4-en-3-one * | 0.01 | ± | 0.05 | 9 |  | - |  |  |
| 64.32 | Stigmasterol * | 0.05 | ± | 0.16 | 9 | 1.05 | ± | 1.38 | 67 |
| 64.80 | Stigmastanol * | 0.49 | ± | 1.10 | 18 | 0.86 | ± | 0.82 | 78 |
| 65.87 | β -Sitosterol * | 0.79 | ± | 1.92 | 18 | 2.89 | ± | 4.75 | 78 |
| 66.14 | Stigmastanol unknown derivative (430) | 0.07 | ± | 0.18 | 18 | 0.38 | ± | 0.70 | 56 |
| 66.80 | Olean-12-en-3β -ol (β-Amyrin) * |  | - |  |  | 0.27 | ± | 0.66 | 22 |
| 67.02 | Germanicol |  | - |  |  | 0.26 | ± | 0.63 | 22 |
| 67.50 | Stigmast-7-en-3-ol | 0.36 | ± | 1.10 | 18 | 0.56 | ± | 1.24 | 44 |
| 68.17 | Urs-12-en-3 β -ol (α-Amyrin) * | 0.01 | ± | 0.02 | 9 | 0.43 | ± | 1.06 | 22 |
| 71.17 | Lup-20(29)-ene-3,28-diol |  | - |  |  | 0.10 | ± | 0.24 | 22 |
|  | |  |  |  |  |  |  |  |  |
| ALKANES: | |  |  |  |  |  |  |  |  |
| 24.04 | Dodecane * | 0.55 | ± | 0.87 | 36 | 0.50 | ± | 1.00 | 22 |
| 27.00 | Tridecane * | 2.41 | ± | 3.14 | 64 |  | - |  |  |
| 29.72 | Tetradecane * | 0.33 | ± | 0.46 | 45 |  | - |  |  |
| 32.15 | Pentadecane * | 0.66 | ± | 1.00 | 54 |  | - |  |  |
| 33.23 | Unidentified branched alkane | 0.61 | ± | 0.88 | 54 |  | - |  |  |
| 34.61 | Hexadecane * | 0.89 | ± | 0.91 | 64 |  | - |  |  |
| 36.87 | Heptadecane * | 0.55 | ± | 0.55 | 64 |  | - |  |  |
| 37.11 | Unidentified branched alkane | 0.77 | ± | 0.85 | 64 |  | - |  |  |
| 38.04 | Unidentified branched alkane | 0.82 | ± | 0.82 | 64 |  | - |  |  |
| 38.99 | Octadecane * | 3.35 | ± | 3.19 | 91 |  | - |  |  |
| 39.19 | Unidentified branched alkane | 0.43 | ± | 0.54 | 45 |  | - |  |  |
| 41.03 | Unidentified branched alkane | 0.23 | ± | 0.44 | 27 |  | - |  |  |
| 41.53 | Unidentified branched alkane | 0.79 | ± | 0.94 | 54 |  | - |  |  |
| 42.13 | Unidentified branched alkane | 0.18 | ± | 0.40 | 18 |  | - |  |  |
| 42.42 | Nonadecane * | 0.69 | ± | 1.12 | 45 |  | - |  |  |
| 42.97 | Eicosane * | 2.92 | ± | 2.90 | 91 | 0.04 | ± | 0.10 | 11 |
| 43.08 | Unidentified branched alkane | 0.51 | ± | 0.65 | 45 |  | - |  |  |
| 43.51 | Unidentified branched alkane | 0.16 | ± | 0.40 | 18 |  | - |  |  |
| 46.28 | Unidentified branched alkane | 0.31 | ± | 0.34 | 54 |  | - |  |  |
| 46.61 | Docosane * | 2.75 | ± | 2.73 | 82 |  | - |  |  |
| 46.97 | Unidentified branched alkane | 0.33 | ± | 0.52 | 36 |  | - |  |  |
| 49.97 | Tetracosane * | 2.14 | ± | 2.09 | 73 |  | - |  |  |
| 51.55 | Pentacosane * | 0.13 | ± | 0.32 | 27 | 0.06 | ± | 0.09 | 56 |
| 53.07 | Hexacosane * | 1.67 | ± | 1.54 | 82 | 0.02 | ± | 0.04 | 33 |
| 53.21 | Unidentified branched alkane | 0.10 | ± | 0.32 | 9 | 0.02 | ± | 0.04 | 22 |
| 53.67 | Unidentified branched alkane | 0.27 | ± | 0.80 | 18 | 0.42 | ± | 0.57 | 56 |
| 54.55 | Heptacosane * | 0.86 | ± | 1.66 | 45 | 1.16 | ± | 1.92 | 56 |
| 55.05 | 11-Methylheptacosane | 0.47 | ± | 1.24 | 18 | 1.37 | ± | 2.55 | 56 |
| 55.28 | Unidentified branched alkane | 0.09 | ± | 0.25 | 18 | 0.02 | ± | 0.04 | 33 |
| 55.42 | Unidentified branched alkane | 0.02 | ± | 0.07 | 9 | 0.02 | ± | 0.03 | 33 |
| 55.73 | Unidentified branched alkane | 0.04 | ± | 0.12 | 9 | 0.20 | ± | 0.40 | 33 |
| 56.05 | Octacosane * | 1.01 | ± | 1.43 | 54 |  | - |  |  |
| 56.78 | Unidentified branched alkane | 0.03 | ± | 0.09 | 9 | 0.07 | ± | 0.17 | 22 |
| 57.35 | Nonacosane * | 1.85 | ± | 3.30 | 45 | 4.86 | ± | 5.29 | 78 |
| 57.82 | 11-Methylnonacosane | 3.53 | ± | 7.37 | 36 | 8.74 | ± | 10.24 | 89 |
| 58.10 | Unidentified branched alkane | 1.95 | ± | 5.76 | 27 | 1.48 | ± | 2.24 | 67 |
| 58.20 | Triacontane * | 0.59 | ± | 1.95 | 9 | 0.99 | ± | 1.94 | 56 |
| 58.55 | Dotriacontane * | 0.45 | ± | 1.42 | 18 | 0.94 | ± | 0.98 | 78 |
| 59.02 | Tritriacontane * | 0.79 | ± | 1.24 | 45 | 0.67 | ± | 1.63 | 11 |
| 60.66 | Tetratriacontane * | 0.84 | ± | 2.78 | 9 |  | - |  |  |
| 62.12 | Pentatriacontane * |  | - |  |  | 0.62 | ± | 0.85 | 44 |
| 62.79 | Hexatriacontane * | 0.11 | ± | 0.37 | 9 |  | - |  |  |
| 65.38 | Unidentified branched alkane | 0.08 | ± | 0.26 | 9 | 0.50 | - | 1.00 |  |
|  | |  |  |  |  |  |  |  |  |
| TERPENOIDS: | |  |  |  |  |  |  |  |  |
| 56.41 | Squalene * | 11.24 | ± | 12.24 | 100 | 10.71 | ± | 19.37 | 100 |
|  | |  |  |  |  |  |  |  |  |
| ALCOHOLS: | |  |  |  |  |  |  |  |  |
| 24.07 | 2-Decanol | 0.02 | ± | 0.06 | 9 | 0.11 | ± | 0.27 | 22 |
| 27.41 | Unidentified alcohol | 0.79 | ± | 1.22 | 54 | 2.05 | ± | 5.03 | 11 |
| 38.35 | Unidentified alcohol | 1.34 | ± | 1.63 | 54 |  | - |  |  |
| 38.53 | Unidentified alcohol | 0.78 | ± | 0.97 | 54 |  | - |  |  |
| 38.79 | Unidentified alcohol | 0.43 | ± | 0.70 | 36 |  | - |  |  |
| 42.62 | Unidentified alcohol | 0.41 | ± | 0.92 | 18 |  | - |  |  |
| 43.24 | Unidentified alcohol | 0.27 | ± | 0.60 | 18 |  | - |  |  |
| 47.38 | Unidentified alcohol | 0.43 | ± | 0.64 | 45 |  | - |  |  |
|  | |  |  |  |  |  |  |  |  |
| AROMATIC HETEROCYCLIC COMPOUNDS | |  |  |  |  |  |  |  |  |
| 26.25 | Indolizine | 0.01 | ± | 0.01 | 9 |  | - |  |  |
| 26.84 | Indole * | 0.01 | ± | 0.04 | 9 | 1.37 | ± | 0.93 | 89 |
| 29.44 | 3-Methyl indolizine | 0.01 | ± | 0.04 | 9 | 0.09 | ± | 0.13 | 56 |
|  | |  |  |  |  |  |  |  |  |
| METHYL ESTERS OF CARBOXYLIC ACIDS: | |  |  |  |  |  |  |  |  |
| 38.77 | Tetradecanoic acid, 12-methyl, methyl ester |  | - |  |  | 0.01 | ± | 0.02 | 33 |
| 38.94 | Pentadecanoic acid, methyl ester * |  | - |  |  | 0.05 | ± | 0.10 | 33 |
| 39.55 | Tetradecanoic acid, 1-methylethyl ester * | 0.14 | ± | 0.33 | 18 |  | ± |  |  |
| 41.16 | 9-Hexadecenoic acid, methyl ester * |  | - |  |  | 0.01 | ± | 0.02 | 33 |
| 41.56 | Hexadecanoic acid, methyl ester * | 0.01 | ± | 0.01 | 9 | 0.02 | ± | 0.04 | 33 |
| 44.76 | (*Z,Z)-*9,12-Octadecadienoic acid, methyl ester * |  | - |  |  | 0.09 | ± | 0.19 | 33 |
| 44.87 | (Z)-9-Octadecenoic acid, methyl ester * | 0.06 | ± | 0.17 | 18 | 0.25 | ± | 0.51 | 33 |
| 44.96 | (E)-9-Octadecenoic acid, methyl ester * |  | - |  |  | 0.01 | ± | 0.02 | 22 |
| 45.66 | 9,15-Octadecadienoic acid, methyl ester |  | - |  |  | 0.01 | ± | 0.02 | 22 |
|  | |  |  |  |  |  |  |  |  |
| ALDEHYDES: | |  |  |  |  |  |  |  |  |
| 39.34 | Hexadecanal * | 0.18 | ± | 0.48 | 36 | 0.04 | ± | 0.08 | 33 |
| 42.92 | An Octadecenal |  | - |  |  | 0.03 | ± | 0.07 | 33 |
| 43.38 | Octadecanal * |  | - |  |  | 0.01 | ± | 0.02 | 33 |
|  | |  |  |  |  |  |  |  |  |
| KETONES: | |  |  |  |  |  |  |  |  |
| 23.82 | 2-Decanone * | 0.02 | ± | 0.05 | 9 | 0.39 | ± | 0.78 | 33 |
|  | |  |  |  |  |  |  |  |  |
| OTHERS | |  |  |  |  |  |  |  |  |
| 43.68 | Cyclic octaatomic sulfur | 0.01 | ± | 0.01 | 9 |  | - |  |  |
